# PCV2-DNA in formalin-fixed and paraffin embedded lymph nodes of wild boar (*Sus scrofa *ssp. *scrofa*): one sampling approach for two laboratory techniques

**DOI:** 10.1186/1751-0147-54-17

**Published:** 2012-03-26

**Authors:** Federico Morandi, Serena Panarese, Ranieri Verin, Fabio Ostanello, Cinzia Benazzi, Giuseppe Sarli

**Affiliations:** 1 Department of Veterinary Medical Science, University of Bologna, via Tolara di Sopra 50, 40064 Ozzano dell'Emilia, Bologna, Italy; 2 Department of Animal Pathology, Prophylaxis and Food Hygiene, University of Pisa, Viale delle Piagge 2, 56124 Pisa, Italy

## Abstract

Superficial inguinal lymph nodes from 72 wild boars examined in a previous immunohistochemical (IHC) study on porcine circovirus type 2 (PCV2) were selected for a PCV2 polymerase chain reaction (PCR) analysis. Four of these lymph nodes were PCV2-IHC strongly positive with PMWS histological lesions (outcome 1), 6 weak to mild PCV2-IHC positive without PMWS histological lesions (outcome 2) and 62 PCV2-IHC negative. Considering IHC the gold standard for diagnosis, the aims of the study were to evaluate the suitability of the PCV2-DNA extraction from formalin-fixed and paraffin-embedded (FFPE) tissue and the sensitivity and specificity of PCR under two IHC interpretations criteria: (A) the sample was considered positive if the result was outcome 1; (B) the sample was considered positive if the result was outcome 1 or 2. Under (A) criteria, sensitivity and specificity of PCR were 100% and 89.7%, respectively; the Cohen's Kappa coefficient was 0.49. Under (B) criteria, sensitivity and specificity of PCR were 80.0% and 95.2%, respectively; the Cohen's Kappa coefficient was 0.72. The high Cohen's Kappa coefficient under the (B) interpretative criteria indicates good agreement between the two methods. In conclusion, 1) DNA extracted from FFPE specimens of wild boar is suitable for PCR and further represents a screening test for PCV2/PCVD (PCV2 Diseases) investigations in wild boar as well; 2) routine histological sampling can also be useful for PCV2 virological studies in wild boar.

## Findings

Since 1998, Porcine Circovirus type 2 (PCV2) has been recognized to play an important role in postweaning multisystemic wasting syndrome (PMWS), as well as in many other pathologies in pig [1] defined as PCVD (PCV2 Diseases), causing huge economic losses to swine husbandry in all affected countries. The host spectrum is limited to the genus *Sus *[1] and numerous studies report in wild boar both PCV2 infection and associated diseases [2-5]. The wide spread of the infection and the absence of a close correlation between this and the pathological description made *in situ *tests (immunohistochemistry-IHC and *in situ *hybridization-ISH) the gold standard for the diagnosis of PCVD [1], whereby the causative agent is highlighted in the lesion. Despite this, it is also true that the greater sensitivity of PCR based methods [6] can provide more accurate information in assessing the infection prevalence in wild boar [3,7,8]. In many cases, paraffin embedded material is available as well as frozen serum samples [9,10].

In the light of that, some studies have developed sophisticated techniques to extract viral nucleic acid, from formalin-fixed and paraffin-embedded (FFPE) specimens, sufficiently preserved to be submitted for biomolecular investigations [10,11]. The objectives of the present study were: 1) to carry out PCV2-DNA extraction and a subsequent investigation on FFPE with PCR in wild boar; 2) to compare the PCR results with those obtained on the same samples by IHC.

In a previous study [2], 148 superficial inguinal lymph nodes, from as many wild boar shot in the Bologna Province (44°00'N, 11°00'E) and in the Colli Euganei Regional Park (45°14'N, 11°45'E), were examined with an IHC technique. Within the total amount, 72 lymph nodes were selected according to the following criteria: PCV2-IHC strongly positive lymph nodes with PMWS histological lesions (4 samples; outcome 1); weak to mild PCV2-IHC positivity without PMWS histological lesions (6 samples; outcome 2); randomly chosen PCV2-IHC negative lymph nodes (62 samples; outcome 3) (Table 1).

**Table 1:** Comparison of PCR and IHC results

		IHC			
		**strongly** **positive** **(outcome 1)**	**mildly/weakly positive** **(outcome 2)**	**negative** **(outcome 3)**	

**PCR**	**+**	4	4	3	11

	**-**	0	2	59	61

		4	6	62	72

A number of FFPE 4-μm-thick sections were cut in order to collect 20 mg of tissue in a 1.5 ml tube. The sections were dewaxed twice in 1.2 ml of Solvent Plus (Carlo Erba, Milan, Italy) at room temperature (RT) for 10 minutes and centrifuged at 13.000 Rpm for 4 minutes. The specimens were then rinsed twice in 1.2 ml of 100% Ethanol (Carlo Erba, Milan, Italy) at RT and centrifuged at 13.000 Rpm for 4 minutes. The tissues were dried at 37°C for 15 minutes (allowing ethanol to evaporate) and then processed for DNA isolation with a RBC BioScience kit, according to the manufacturer's instructions. Nucleic acid was then loaded on a PCR reaction in accordance with the protocol published by Ouardani et al. [12]. The set of primers, including ORF2.PCV2.S4 and ORF2.PCV2.AS4, was designed to amplify a 493 bp product located on PCV2 ORF-2. The cycling conditions were the following: 1' at 95°C; 1' at 95°C, 1' at 55°C, 1' at 72°C (for 35 cycles); a final extension at 72°C for 7'. Products were run on a 1% agarose gel, in 1x TAE Buffer, stained with GelRed and DNA fragments were separated by size by electrophoresis. The relative sensitivity and specificity of PCR versus IHC were evaluated using two criteria of interpretation: A) considering positive only samples with "outcome 1" (strict interpretation), or B) considering positive samples with "outcome 1 or 2" (permissive interpretation). The overall agreement between the two methods, separately as for strict and permissive interpretation, was calculated as well as sensitivity and specificity.

The results from the 72 samples are presented in Table 1. Comparing the two methods, an overall agreement of results (IHC vs PCR) was found in 93.1% of cases (67 of 72). Concordance was 100% in the 4 cases showing both typical PMWS lesions and strong IHC positivity (IHC-outcome 1). In 2 samples, showing moderate to weak IHC positivity and focal distribution (IHC-outcome 2), PCR was negative. The opposite result was registered in 3 subjects of the IHC negative group (IHC-outcome 3) that showed PCR positivity. Finally, 10 of the 72 selected samples were IHC-positive, while PCR revealed a ratio of 11/72 positive. For IHC strict interpretation, sensitivity and specificity of PCR vs IHC were 100% and 89.7% (95% C.I.: 82.5-96.9), respectively. For IHC permissive interpretation, they were 80.0% (95% C.I.: 55.2-100) and 95.2% (95% C.I.: 89.8-100), respectively. Cohen's Kappa values were 0.49 (moderate agreement) and 0.72 (good agreement), respectively.

The mutual importance of the domestic pig and wild boar in the spread and transmission of PCV2 has been recently investigated [3]. The availability of methods to conduct retrospective studies in pigs [9,13] can help to increase knowledge regarding the dynamics of PCV2 infection also in wild boar. By means of the proposed techniques, it is possible to extract PCV2-DNA from FFPE samples, as in domestic pigs, both in subjects bearing PMWS lesions and in those with only infection.

The method can provide several advantages such as: 1) the use of archival material for retrospective studies on the epidemiology of PCV2 infection in the wild; 2) histological sampling to be also tested and preserved for DNA assessments (simplifying the work of both technicians and hunters, as one sample can cover two fields of investigation). It is well known that DNA quality from FFPE specimens depends on many factors, such as the length of time between surgical removal of the tissues and formalin fixation, prolonged storage of samples (in formalin or in paraffin blocks) [10,11], variable levels of nucleases detected in different tissues [14]. Both the results of amplification and IHC can be affected by subcellular modifications (i.e. crosslinking between proteins and/or nucleic acids that provides a strong steric hindrance creating an intricate physical barrier) induced by these treatments [15]. To follow the behavior of our samples, both negative/positive and internal controls were added to the collection examined. As reported in the literature [6], the results show a greater specificity of IHC compared to PCR in PMWS cases, where a very high amount of PCV2 is present in the lesions [1]. Two IHC-positive samples werenegative with PCR and this can be due, as well as to the processing procedure [10,11] also to factors intrinsic to the sample: weakly-positive IHC or, in particular, focal positivity in a few lymphoid follicles, decreasing the probability to select areas containing the virus. Finally, 3 IHC-negative but PCR-positive subjects would confirm the greater sensitivity of the latter technique. However, it should be noted that IHC does not detect only the mere presence of infection, but also proves the presence of the virus within the lesions, allowing the diagnosis of PCVD. In epidemiologic studies in wild pigs, the use of IHC as a single method could represent a limiting factor due to the lower viral load observed than in domestic pigs [3]. This evidence is also supported by a very low prevalence of disease [2-5], suggesting that a more sensitive experimental approach, as the nested-PCR, could allow to achieve more satisfactory results, as also reported by Kim and Chae [10]. The relatively high Cohen's Kappa values observed, especially by permissive interpretation, suggested possible advantages of using the PCR method on FFPE specimens as a possible screening approach also for wild boar samples: because it is more sensitive than IHC, all cases of the disease were diagnosed as positive.

In conclusion, this sampling approach can be adopted as a "ready to use protocol" by field technicians and/or it allows the exploitation of archived samples for epidemiological study of PCV2 in wild boar.

## Competing interests

The authors declare that they have no competing interests.

## Authors' contributions

FM participated in the design of the study, collection of samples and prepared the manuscript. SP carried out lab assessments. RV participated in the design of the study and the collection of samples. FO participated in the analysis data and drafted the manuscript. CB and GS conceived the study, and participated in its design and coordination. All authors read and approved the final manuscript.
